# Activity-dependent endogenous taurine release facilitates excitatory neurotransmission in the neocortical marginal zone of neonatal rats

**DOI:** 10.3389/fncel.2014.00033

**Published:** 2014-02-10

**Authors:** Taizhe Qian, Rongqing Chen, Masato Nakamura, Tomonori Furukawa, Tatsuro Kumada, Tenpei Akita, Werner Kilb, Heiko J. Luhmann, Daiichiro Nakahara, Atsuo Fukuda

**Affiliations:** ^1^Department of Neurophysiology, Hamamatsu University School of MedicineHamamatsu, Japan; ^2^Institute of Physiology, University Medical Center of the Johannes Gutenberg-UniversityMainz, Germany; ^3^Department of Psychology and Behavioral Neuroscience, Hamamatsu University School of MedicineHamamatsu, Japan; ^4^Department of Occupational Therapy, Tokoha UniversityHamamatsu, Japan

**Keywords:** taurine, GABA_A_ receptor, glycine receptor, marginal zone, NKCC1, GABA, microdialysis

## Abstract

In the developing cerebral cortex, the marginal zone (MZ), consisting of early-generated neurons such as Cajal-Retzius cells, plays an important role in cell migration and lamination. There is accumulating evidence of widespread excitatory neurotransmission mediated by γ-aminobutyric acid (GABA) in the MZ. Cajal-Retzius cells express not only GABA_A_ receptors but also α2/β subunits of glycine receptors, and exhibit glycine receptor-mediated depolarization due to high [Cl^−^]_i_. However, the physiological roles of glycine receptors and their endogenous agonists during neurotransmission in the MZ are yet to be elucidated. To address this question, we performed optical imaging from the MZ using the voltage-sensitive dye JPW1114 on tangential neocortical slices of neonatal rats. A single electrical stimulus evoked an action-potential-dependent optical signal that spread radially over the MZ. The amplitude of the signal was not affected by glutamate receptor blockers, but was suppressed by either GABA_A_ or glycine receptor antagonists. Combined application of both antagonists nearly abolished the signal. Inhibition of Na^+^, K^+^-2Cl^−^ cotransporter by 20 µM bumetanide reduced the signal, indicating that this transporter contributes to excitation. Analysis of the interstitial fluid obtained by microdialysis from tangential neocortical slices with high-performance liquid chromatography revealed that GABA and taurine, but not glycine or glutamate, were released in the MZ in response to the electrical stimulation. The ambient release of taurine was reduced by the addition of a voltage-sensitive Na^+^ channel blocker. Immunohistochemistry and immunoelectron microscopy indicated that taurine was stored both in Cajal-Retzius and non-Cajal-Retzius cells in the MZ, but was not localized in presynaptic structures. Our results suggest that activity-dependent non-synaptic release of endogenous taurine facilitates excitatory neurotransmission through activation of glycine receptors in the MZ.

## Introduction

The marginal zone (MZ) is an important layer located at the surface of the developing neocortex that will later develop into the cell-sparse layer I (Fairen et al., 2002). However, in the first postnatal week of rodent development, it is densely populated with several early generated neuronal populations with Cajal-Retzius cells representing the main cell type in the MZ (Larroche, 1981; Frotscher, 1998; Marín-Padilla, 1998; Jiménez et al., 2003; Janusonis et al., 2004; Soriano and Del Río, 2005). These cells produce the extracellular matrix protein reelin, which regulates neuronal migration and affects axonal growth and synaptogenesis in the developing cortex (D’Arcangelo et al., 1995; Hirotsune et al., 1995; Aguiló et al., 1999; Borrell et al., 1999; Radnikow et al., 2002; Frotscher et al., 2009; Sekine et al., 2012; Gil-Sanz et al., 2013). Thus, the MZ plays an important role in cell migration and lamination of the developing cerebral cortex. Although Cajal-Retzius cells are transient cell populations, both Cajal-Retzius cells and non-Cajal-Retzius cells in the MZ receive functional synaptic inputs and are integrated into transient synaptic circuits (Hestrin and Armstrong, 1996; Zhou and Hablitz, 1996; Radnikow et al., 2002; Luhmann et al., 2003; Soda et al., 2003). The synaptic integration of these cell types, with their essential role in structural development, may represent one essential factor for the activity-dependent maturation of the neocortex (Kilb et al., 2011).

The MZ receives mainly excitatory GABAergic inputs during early postnatal development (Mienville, 1998; Schwartz et al., 1998; Aguiló et al., 1999; Dammerman et al., 2000a; Kilb and Luhmann, 2001; Soda et al., 2003; Achilles et al., 2007; Kirmse et al., 2007; Kolbaev et al., 2011b). In addition, we reported glycine receptor-mediated depolarization and functional expression of α2/β glycine receptor subunits in Cajal-Retzius cells (Kilb et al., 2002; Okabe et al., 2004). Because no evidence for synaptic glycinergic transmission has been observed, it was suggested that in the immature cortex glycine receptors are activated by extrasynaptic ligands, presumably by taurine (Flint et al., 1998; Kilb et al., 2008). Although it has been shown that glycine receptors can influence neuronal migration in the developing cerebral cortex (Nimmervoll et al., 2011), the physiological role of glycine receptors for neurotransmission in the MZ and their relationship to early GABAergic inputs is not well understood. Because GABAergic fibers project to the MZ (Lin et al., 1990; Dammerman et al., 2000a) and glycine receptors are strongly expressed in the MZ before birth (Malosio et al., 1991), their interaction may influence cortical development through synaptic contacts of Cajal-Retzius cells and other cells in the MZ. Thus, physiological roles of endogenous glycine receptor agonists in the neurotransmission of the MZ need to be elucidated.

In the present study, optical imaging by means of voltage-sensitive dyes as well as whole-cell patch-clamp recordings were employed to monitor neurotransmission in the MZ of neonatal rat brain slices. In addition, microdialysis followed by high-performance liquid chromatography (HPLC) analyses was performed to investigate neurotransmitter release. We demonstrated that the propagation of electrically evoked activity in the MZ is mainly mediated by GABA_A_ and glycine receptors and that repetitive stimulation increased the extracellular GABA and taurine levels. The results suggest that endogenous taurine facilitates excitatory neurotransmission in the neonatal MZ.

## Materials and methods

All experiments were performed in accordance with EU directive 86/609/EEC for the use of animals in research, and conformed to the guidelines on the ethical use of animals for animal experimentation at Hamamatsu University School of Medicine and the Johannes Gutenberg University Mainz (approved by the Landesuntersuchungsanstalt RLP, Koblenz, Germany). All efforts were made to minimize the number of animals used and their suffering.

### Preparation of tangential brain slices

Tangential slices of the neocortex were prepared as described previously (Kilb and Luhmann, 2000). In brief, neonatal Wistar rats (Japan SLC, Shizuoka, Japan) at postnatal day (P) P0–P3 were deeply anesthetized by hypothermia and decapitated. The brain was quickly removed and stored for 1–2 min in ice-cold artificial cerebrospinal fluid (ACSF). The solution contained the following (mM): 220 sucrose, 2.5 KCl, 1.25 NaH_2_PO_4_, 12.0 MgSO_4_, 0.5 CaCl_2_·2H_2_O, 26.0 NaHCO_3_, 30.0 glucose. Hemispheres were dissected at the midline, the pia mater was removed, and tangential slices were cut at a maximal thickness of approximately 400 µm containing the MZ. The slices were subsequently mounted on fine tissue paper and were allowed to recover for at least 60 min in standard ACSF consisting of (mM): 126 NaCl, 2.5 KCl, 1.25 NaH_2_PO_4_, 2.0 MgSO_4_, 2.0 CaCl_2_, 26.0 NaHCO_3_ and 20.0 glucose. Slices were either placed in a tightly sealed box filled with 95% O_2_–5% CO_2_ at a pressure of 50 kPa at room temperature (Fukuda and Prince, 1992) or kept in an incubation chamber with ACSF saturated with 95% O_2_–5% CO_2_ at room temperature.

### Optical recording with voltage-sensitive dye

Tangential neocortical slices were incubated with the voltage-sensitive dye JPW1114 (100 µM) for 30 min. The paper-mounted slices were then transferred into a submerged recording chamber (volume *ca*. 1 mL) attached to a fixed stage of an upright microscope (E600-FN, Nikon, Tokyo, Japan) and were superfused with standard ACSF at a rate of 1–2 mL/min. A bipolar tungsten electrode (World Precision Instruments, Sarasota, FL, USA) was used to evoke synaptic transmission in the tangential neocortical slice. Synaptic responses were evoked by a current pulse of 1 mA with 100 µs duration delivered at 1 Hz. Eight consecutive responses were averaged for analysis. All experiments were performed at 30 ± 0.5^°^C.

A 24-V/300-W tungsten-halogen lamp was used for excitation of the voltage-sensitive dye. After slices were viewed with a 4× objective lens (N. A. 0.10; Nikon), optical recording was achieved by using a 10× water immersion objective lens (N.A. 0.30; Nikon) and filter cube comprised of a 510–560 nm excitation filter, a 570 nm dichroic mirror, and a 590 nm barrier filter. Emitted fluorescence was detected by a high-speed 80 × 80 pixel back-thinned charge-coupled device (CCD) camera (NeuroCCD, Red Shirt Imaging, Fairfield, CT, USA). Photocurrents were converted to voltages and were DC-coupled to analog-to-digital converters using a 16-bit resolution at a frame rate of 1 kHz. Off-line data analysis was performed using the NeuroPlex software (Red Shirt Imaging).

Dye bleaching was corrected off-line and light intensities were measured as relative fluorescence change (Δ*F*/*F*), where *F* is the fluorescent light intensity of the stained slice during illumination without evoked neuronal activity and Δ*F* is the fluorescence change during neuronal activity. A decrease in fluorescence (plotted upwards in all figures) corresponded to membrane depolarization, and an increase in fluorescence plotted downwards corresponded to membrane hyperpolarization.

### Microdialysis-HPLC measurement

A probe for microdialysis was inserted into the tangential neocortical slice at approximately 800 µm apart from the stimulating electrode. The microdialysis collections started more than 60 min before stimulation began. The procedure for microdialysis was described previously (Nakahara et al., 2001). In brief, the dialysis probe was perfused with the standard ACSF at a flow rate of 2 µL/min. Measurements of steady-state levels of amino acids began after a 60-min stabilization period. Three consecutive samples were collected at 20-min intervals in small plastic vials to determine steady-state levels, followed by six consecutive samples to establish temporal changes induced by electrical stimulation. After samples were derivatized with *o*-phthalaldehyde, the concentrations of amino acid neurotransmitters including GABA, glutamate, glycine and taurine were determined by reverse-phase HPLC with electrochemical detection. The HPLC system (BAC-300 system, EICOM, Kyoto, Japan) consisted of an EP-300 pump, an ECD-300 electrode, and a PowerChrom system (ADI, Sydney, Australia). Separation of amino acid derivatives was achieved using an Eicompack MA-5ODS column. The detection potential was set at +700 mV against an Ag/AgCl reference electrode. The flow rate was 1.20 mL/min, and the sensitivity was set at 64 nA/V full-scale. The mobile phase consisted of 100 mM phosphate buffer (pH 6.0) and 30% (v/v) methanol. The amino acids and the derivatives were mixed and allowed to react for exactly 2 min before injection.

### Immunohistochemistry

Animals were perfusion-fixed with 4% (w/v) paraformaldehyde and 0.5% (v/v) glutaraldehyde in 0.1 M phosphate-buffered saline (PBS) and brains removed were post-fixed with the same fixative. The sections were cut at 20 µm on a cryostat, thaw-mounted onto silane-coated slides. The sections were pretreated with 0.5% (w/v) sodium borohydride for 30 min. Sections were first treated in 0.3% (v/v) H_2_O_2_ for 30 min. After washing with PBS, the sections were incubated with a blocking solution (10% (v/v) normal goat serum, 0.1% (v/v) Triton X-100 in PBS) for 1 h at room temperature. Sections were then incubated at 4^°^C for 36 h with rabbit anti-taurine polyclonal antibody (TT100; 1:100; Signature Immunologics, Salt Lake City, UT, USA), for 1 h with biotinylated goat anti-rabbit IgG (1:100; Vector Laboratories, Burlingame, CA, USA). After rinsing in 0.1 M PBS, the sections were incubated with avidin–horseradish peroxidase (Vectastain ABC kit; Vector Laboratories) for 30 min at room temperature. After washing with PBS, the immunoreaction was developed for 2–3 min with 0.01% (w/v) 3,3′-diaminobenzidine (Sigma-Aldrich, St. Louis, MO, USA) activated by 0.01% (v/v) H_2_O_2_ .

### Electron microscopy

After the animals (P0) were deeply anesthetized with halothane and perfused transcardially with 4% (w/v) paraformaldehyde/0.5% (v/v) glutaraldehyde in 0.1 M HEPES buffer, the brains were removed and post fixed for 10 min. Coronal sections were cut at 50 µm with a vibratome (DTK-1500; Dosaka, Kyoto, Japan) in the same fixative solution. Sections were kept in 0.1 M HEPES buffer for 1 day at 4^°^C. After rinsing in PBS, sections were incubated for 36 h at 4^°^C with polyclonal antibody against taurine (Signature Immunologics). The sections were washed with PBS several times, and then treated with anti-rabbit secondary antibody coupled with 1.4-nm-diameter gold particles (Nanogold; Nanoprobes, Yaphank, NY, USA) overnight at room temperature. Immunoelectron microscopic analysis was performed by Tokai-EMA (Nagoya, Japan). After the sections were washed with PBS and fixed in 2% (v/v) glutaraldehyde in 0.1 M sodium cacodylate buffer (pH 7.4) for 3 h at room temperature, gold particles were enlarged for microscopic examination with the HQ-Silver Enhancement Kit (Nanoprobes) and routinely processed for electron microscopic examination. The embedded samples were sectioned (thickness 80 nm) with an ultra-microtome (LKB2088; LKB, Bromma, Sweden) and stained with uranyl acetate and lead citrate. Sections were then carbon-coated in a vacuum and observed with a JEOL transmission electron microscope (JEM 2000EX; JEOL, Tokyo, Japan) at 100 kV.

### Electrophysiology

Recordings were obtained at 31 ± 1^°^C from visually identified Cajal-Retzius cells in tangential slices using infrared differential interference contrast (DIC) video microscopy. Neurons and electrodes were visualized by means of an upright microscope equipped with DIC optics (Axioskop 2 FS plus; Zeiss, Jena, Germany), an infrared (IR) filter and a video system (Hamamatsu Photonics, Hamamatsu, Japan). Recording electrodes were pulled from borosilicate tubes (GC200F; Science Products, Hofheim, Germany) using a vertical puller (PP430, Narishige, Tokyo, Japan) and were filled with (in mM): 80 K-gluconate, 44 KCl, 2 MgCl_2_, 1 CaCl_2_, 11 EGTA, 10 K-HEPES, 2 Na_2_-ATP, 0.5 Na-GTP (pH adjusted to pH 7.4 with KOH and osmolarity to 306 ± 2 mOsm with sucrose). Electrode resistance was 4–5 MOhm. Recordings were performed using a discontinuous patch-clamp amplifier (SEC05-L; NPI, Tamm, Germany). Signals were amplified and low-pass-filtered at 3 kHz, digitized online with an AD/DA-board (ITC-16; Heka, Lambrecht, Germany), and analyzed off-line using WinTida software (Heka). All potentials were corrected *post-hoc* for a liquid junction potential of 9.6 mV. Action potential amplitude was determined from the difference between action potential threshold and peak voltage. Synaptic inputs were evoked by monopolar electrical impulses (1 mA, 100 µs) applied by bipolar tungsten electrodes (FHC, Bowdoinham, ME, Impedance 4–5 MOhm) using an A365 (WPI, Sarasota, FL) stimulation unit.

### Drugs

The following drugs were used: JPW1114 (also named di-2 ANEPEQ) (Molecular Probes/Life Technology, Carlsbad, CA, USA); bicuculline methiodide (BIC) and strychnine (STR) (Sigma-Aldrich); 6-cyano-7-nitroquinoxaline-2,3-dione (CNQX), DL-2-amino-5-phosphono-pentanoic acid (D-AP5), picrotoxin (PTX), bumetanide (BUM) and 2-(guanidino)ethanesulfonic acid (GES) (Tocris Bioscience, Bristol, UK); tetrodotoxin (TTX) (Wako, Tokyo, Japan).

### Statistics

Unless otherwise indicated, all numerical data are shown as the mean ± the standard error of the mean (SEM). Differences between two groups were assessed using Student’ s paired *t*-test for absolute and Mann–Whitney *U*-test for normalized values. Comparisons among several groups were performed using one-way analysis of variance (ANOVA) with *post-hoc* Tukey test. In the microdialysis study, the Friedman test was used to evaluate the overall response with time. A *P* < 0.05 was considered statistically significant.

## Results

### Optical voltage recordings of tangential slice preparations from the marginal zone (MZ)

**Figure 1:**
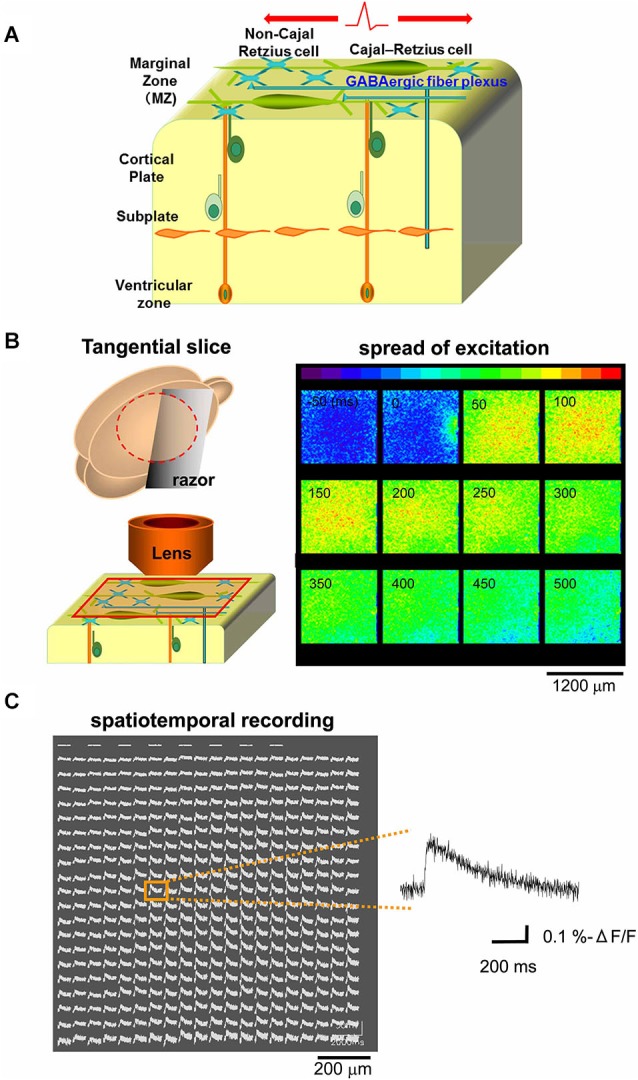
**Optical voltage recording in tangential neocortical slices**. **(A)** Schematic diagram showing cellular and axonal components of the tangential direction of the MZ. **(B)** Left: schematic diagram showing preparation of tangential slice and optical recording from the MZ. Right: the main component of the optical signal evoked by single electrical stimulations spread out over the MZ, so that excitatory optical signals were recorded spatially (colored in red). Note that the stimulating electrode was placed on the edge of the recorded area (see panel at 0 ms). **(C)** Spatiotemporal recordings enable us to analyze the evoked optical signals which spread in horizontal direction within the MZ. Representative traces were chosen (inset) for analyses at distances of 800 µm from the stimulation sites.

Because excitatory GABAergic neurotransmission was reported in the developing MZ (Mienville, 1998; Schwartz et al., 1998; Aguiló et al., 1999; Dammerman et al., 2000a; Kilb and Luhmann, 2001; Soda et al., 2003; Achilles et al., 2007; Kirmse et al., 2007), we hypothesized that excitatory propagation mediated by the GABAergic system could be detected along the tangential direction of the MZ (Figure 1A). Optical signals from the MZ in tangential neocortical slices stained with the fluorescent voltage-sensitive dye JPW1114 were monitored with a high-speed CCD camera. Potentials evoked by single electrical stimulations on the edge of the recorded area spread out rapidly over the MZ monitored (Figure 1B). The spread of excitation was also apparent by spatiotemporal recordings of optical signals (−Δ*F*/*F*) in each slice, of which representative traces were analyzed (Figure 1C).

### Optical signals evoked by electrical stimulation depend on action potentials and are mediated by GABA_A_ glycine receptors

**Figure 2:**
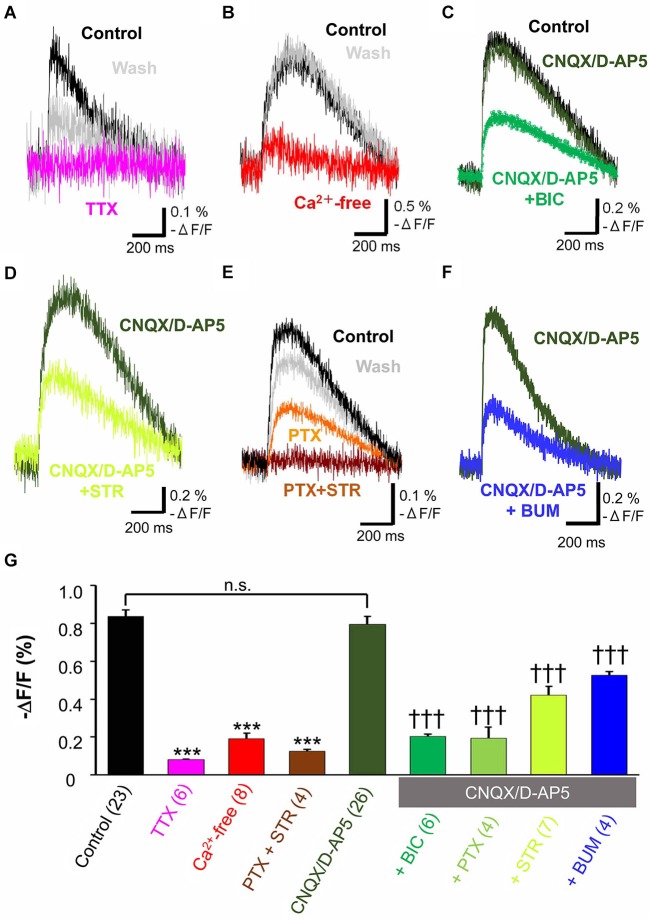
**Pharmacological properties of propagating excitatory optical signals evoked by single electrical stimulation**. To normalize responses, changes in fluorescence intensity were divided by the baseline fluorescence intensity (−Δ*F*/*F*). **(A)** Typical traces of voltage sensitive dye signals before and after treatment with TTX (1 µM). Note that the evoked optical signals were completely blocked by TTX (magenta trace) and partially recovered after washing (grey trace). **(B)** Ca^2+^-free ACSF largely blocked the evoked signals (red trace). **(C)** Evoked optical signals were not affected by combined application of CNQX (10 µM) and D-AP5 (50 µM) (dark green trace). By contrast, the residual signal insensitive to glutamate receptor antagonists was inhibited by the GABA_A_ receptor antagonist BIC (50 µM) (green trace). **(D)** The evoked optical signals were reduced by the glycine receptor antagonist STR (50 µM) (light green trace). **(E)** The evoked optical signals were attenuated by PTX (50 µM), another GABA_A_ receptor-channel blocker (orange trace). Subsequent addition of 50 µM STR further inhibited it down to baseline intensity (brown trace). The optical signal recovered after washing for 30 min (gray trace). **(F)** Evoked optical signals insensitive to glutamate receptor antagonists, CNQX and D-AP5, were inhibited by application of a Na^+^, K^+^-2Cl^−^ cotransporter inhibitor BUM (20 µM) (blue trace). Electrical stimulation-evoked optical signals were recorded after BUM was present at least for 20 min. **(G)** Statistical analysis of the pharmacological profile of propagating excitatory optical signals evoked by single electrical stimulation. The spread of excitation was blocked by TTX and in Ca^2+^-free ACSF. Combined application of PTX and STR also completely abolished the spread of excitation. In contrast, CNQX plus D-AP5 failed to affect the optical signals. Note that the optical signals in the presence of CNQX plus D-AP5 were significantly inhibited by further addition of BIC, PTX or STR (one-way ANOVA, *P* < 0.001, *Post-hoc* Tukey test; ^***^P < 0.001 (vs. control), ^†††^*P* < 0.001 (vs. CNQX + D-AP5), n.s., not significant.). Addition of BUM also attenuated the spread of excitation. Number of trials in parenthesis. Bars represent the mean ± SEM.

When the tangential slices were superfused with 1 µM TTX, a voltage dependent sodium channel blocker, the optical signal disappeared almost completely (11.5 ± 0.8% control, *n* = 6, *P* < 0.001, paired *t*-test; Figure 2A). Ca^2+^-free ACSF also significantly depressed the evoked optical signals (20.8 ± 2.6% control, *n* = 8, *P* < 0.001, paired *t*-test; Figure 2B). These results indicated the spread of the optical signals evoked by electrical stimulation in the MZ depended on action potentials which trigger Ca^2+^-dependent mechanism.

To investigate the neurotransmitter receptors responsible for the propagation of the evoked signals, specific antagonists of amino acid neurotransmitter receptors were used. The combined application of CNQX (10 µM), a non-NMDA receptor antagonist, and D-AP5 (50 µM), an NMDA receptor antagonist, did not affect the evoked optical signals (91.0 ± 3.4% control, *n* = 5, *P* = 0.062, paired *t*-test; Figure 2C). Thus, the spread of excitation was not mediated by glutamate receptors.

Further addition of the GABA_A_ receptor antagonist, BIC (50 µM), significantly reduced the peak optical signal value (29.9 ± 1.8% CNQX/D-AP5, *n* = 6, *P* < 0.001, paired *t*-test; Figure 2C). PTX (50 µM), another GABA_A_ receptor antagonist that blocks GABA_A_ receptor-coupled Cl^−^ channels, also suppressed the evoked optical signals insensitive to glutamate receptor antagonists (20.0 ± 5.5% CNQX/D-AP5, *n* = 4, *P* < 0.05, paired *t*-test), indicating that GABA_A_ receptor activation was involved in the propagation of evoked signals.

When the glycine receptor antagonist STR (50 µM) was added to ACSF containing CNQX and D-AP5, the peak optical signal values were significantly reduced (56.4 ± 0.5% CNQX/D-AP5, *n* = 7, *P* < 0.001, paired *t*-test; Figure 2D), suggesting that glycine receptors were also involved in the propagation of evoked signals. Thus, the spread of excitation in the MZ was mediated not only by GABA_A_ receptors, but also by glycine receptors.

To determine whether GABA_A_ and glycine receptors are differentially activated by endogenous agonists, we examined the additive effects of both antagonists. Figure 2E shows that PTX partially suppressed the evoked optical signals, and that a subsequent addition of STR further suppressed the signals, indicating that both GABA_A_ and glycine receptors were activated. Interestingly, the evoked optical signals were almost completely suppressed by the combined application of PTX and STR even in the absence of CNQX and D-AP5 (14.1 ± 1.2% control, *n* = 4, *P* < 0.001, paired *t*-test; Figure 2E), indicating that co-activation of GABA_A_ and glycine receptors, but not glutamate receptors, should be responsible for the synaptic propagation of evoked optical signals in the MZ. These results suggest that the spread of the optical signals evoked by electrical stimulation in the MZ is attributable to evoked release of GABAergic and glycinergic agonists.

### Cl^−^ uptake by the Na^+^, K^+^-2Cl^−^ cotransporter renders GABA_A_ and glycine receptor- mediated spread of excitation

Previous reports have shown that GABA and glycine responses in Cajal-Retzius cells are excitatory (Kilb et al., 2002; Achilles et al., 2007; Kolbaev et al., 2011a). This effect in immature neurons depends on the Na^+^, K^+^-2Cl^−^ cotransporter. This transporter allows the accumulation of Cl^−^ in cells and contributes to the high intracellular Cl^−^ concentration ([Cl^−^]_i_) found in immature neurons in which GABA_A_ receptor activation is depolarizing and excitatory (Shimizu-Okabe et al., 2002; Payne et al., 2003; Yamada et al., 2004; Dzhala et al., 2005; Fukuda, 2005). Therefore we next used BUM to inhibit Na^+^, K^+^-2Cl^−^ cotransporter and reduce [Cl^−^]_i_.

BUM (20 µM), in the presence of CNQX (10 µM) and D-AP5 (50 µM), significantly reduced the peak optical signal values (59.3 ± 0.7% CNQX/D-AP5, *n* = 4, *P* < 0.001, paired *t*-test; Figure 2F). The result suggested that the Na^+^, K^+^-2Cl^−^ cotransporter contributed to the excitatory neurotransmission in the MZ by increasing [Cl^−^]_i_ higher than a passive distribution, so that GABA_A_ and glycine receptors could mediate membrane depolarization.

Pharmacological properties of the spread of excitation in the MZ were further statistically analyzed by one-way ANOVA followed by *post-hoc* Tukey tests (*P* < 0.0001: ACSF vs. TTX, Ca^2+^-free and PTX + STR, *P* < 0.0001: CNQX/D-AP5 vs. BIC, PTX, STR and BUM) and are summarized in Figure 2G. These analyses supported our previous observations, confirming that electrical stimulation induced TTX-sensitive, Ca^2+^-dependent voltage transients that were independent of glutamate signaling but mediated by depolarizing GABA and glycine responses.

### Electrophysiological responses of single Cajal-Retzius cells upon electrical stimulation

**Figure 3:**
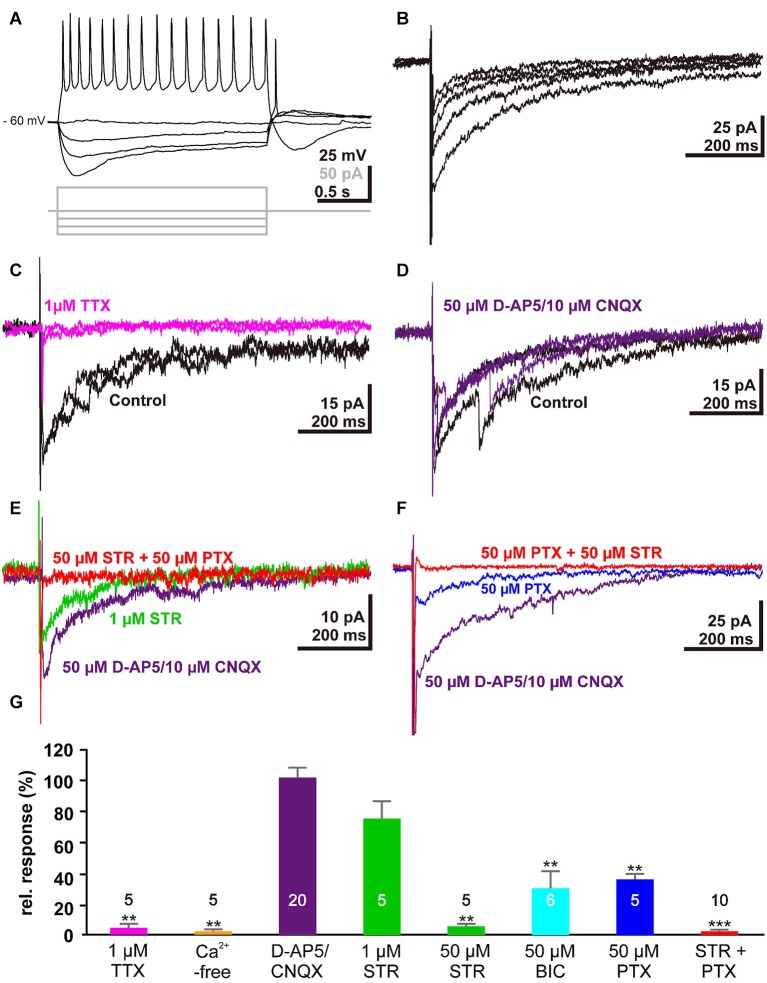
Electrophysiological responses of single Cajal-Retzius cells to electrical stimulation: **(A)** Voltage traces illustrating the typical response of a Cajal-Retzius cell upon injection of de- and hyper-polarizing current pulses. **(B)** Five consecutive current-traces illustrating typical inward current evoked by electrical stimulation in a tangential slice. **(C)** The stimulus-evoked inward current was abolished in the presence of 1 µM TTX (magenta trace). **(D)** Two typical current traces recorded from the same trial illustrating that the stimulus-induced inward current is unaffected in the presence of 50 µM D-AP5 and 10 µM CNQX (purple trace). **(E)** The stimulus-induced inward current was reduced in the presence of 1 µM STR (green trace) and was abolished when both GABA_A_ and glycine receptors were blocked with 50 µM STR and 50 µM PTX, respectively (red trace). **(F)** The stimulus-induced inward current was reduced in the presence of 50 µM PTX (blue trace). **(G)** Statistical analysis. Bars represent the mean ± SEM, numbers of experiments are given in the bars.

To verify that membrane responses in Cajal-Retzius cells underlie the observed alterations in the voltage-sensitive dye signals, we next performed whole-cell patch-clamp recordings from Cajal-Retzius cells in tangential slice preparations. For this purpose we recorded from in total 33 visually identified Cajal-Retzius cells. These cells had an average resting membrane potential of −52.3 ± 6.8 mV (*n* = 33), an input resistance of 1.7 ± 0.2 GΩ (*n* = 33), and responded to depolarization above a threshold of −43.5 ± 0.9 mV (*n* = 33) with action potentials of 42.7 ± 2.2 mV amplitude (*n* = 33) at a maximal frequency of 19.3 ± 1.6 Hz (*n* = 29; Figure 3A), in accordance with previous results (Kilb and Luhmann, 2000, 2001; Sava et al., 2010). Electrical stimulation (1 mA, 100 µs) with a bipolar electrode placed laterally in the tangential slices induced a substantial inward current of 33.2 ± 7.3 pA (*n* = 32) (Figure 3B). This inward current was virtually abolished by 1 µM TTX (0.8 ± 0.5 pA, *n* = 5, Figure 3C, G) and in the presence of Ca^2+^-free solutions (1.4 ± 0.9 pA, *n* = 5; Figure 3G), indicating that action potential and Ca^2+^-dependent processes mediated the observed current. The current was not significantly (*P* = 0.563, Mann–Whitney *U*-test) affected in the combined presence of 10 µM CNQX and 50 µM D-AP5 (38 ± 11.3 pA, corresponding to 102 ± 6% of the control experiments, *n* = 20, Figure 3D, G), indicating that glutamatergic receptors did not contribute to this signal. Therefore, further experiments were performed in the continuous presence of D-AP5/CNQX to block ionotropic glutamate receptors.

The stimulus-induced inward current was reduced by 24 ± 11% (*n* = 5; *P* = 0.095, Mann–Whitney *U*-test) in the presence of 1 µM STR (Figure 3E, G), while in the presence of 50 µM STR the current was significantly (*P* = 0.0053, Mann–Whitney *U*-test) reduced by 95.3 ± 1.6% (*n* = 5). By contrast, 50 µM BIC or 50 µM PTX (Figure 3F, G) reduced the inward current by only 69.6 ± 11% (*n* = 6, *P* = 0.0053, Mann–Whitney *U*-test) or 63.8 ± 3.4% (*n* = 5, *P* = 0.0053, Mann–Whitney *U*-test), respectively. In the combined presence of 50 µM STR and 50 µM PTX the inward current was completely blocked (98.2 ± 0.6 %, *n* = 10, *P* < 0.001, Mann–Whitney *U*-test, Figure 3E, F, G). In summary, these results indicated that the stimulus-induced current was mediated by GABA_A_ and glycine receptors, and that this synaptic response was mediated by agonists acting on both receptors.

### Evoked release of GABA and taurine by focal electrical stimulation

**Figure 4:**
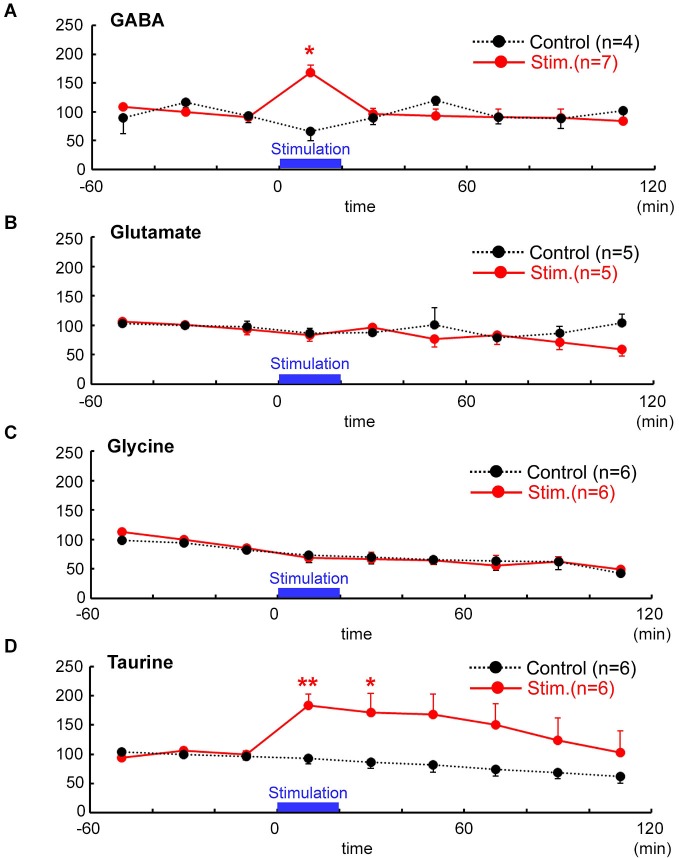
**Temporal changes in neuro-active amino acid concentrations after focal electrical stimulation assessed by microdialysis followed by HPLC**. Evoked release (red) of GABA **(A)** glutamate **(B)** glycine **(C)** and taurine **(D)** were estimated and are indicated as % baseline value. In controls (black), no stimulation was applied. Time after the stimulation is indicated in abscissa (min). Electrical stimulation of 1 mA for 100 µs was delivered at 1 Hz for 20 min, the periods of which are indicated by bars. Baseline values were defined as the average value 60 min immediately before stimulation. Note that taurine and GABA (with 1 mM nipecotic acid), but neither glycine nor glutamate, were significantly increased in response to the electrical stimulation in the MZ. ^**^ *P* < 0.01, ^*^ *P* < 0.05 as compared with control (Mann–Whitney *U*-test).

To examine whether focal electrical stimulation that caused the spread of excitation over the MZ could affect release of endogenous agonists of GABA_A_ and glycine receptors, we monitored the release of GABA, glycine, and taurine besides glutamate from the tangential neocortical slices containing the MZ using microdialysis followed by HPLC. The results are shown in Figure 4. The extracellular concentration of GABA significantly increased from the baseline (1.07 ± 0.07 pmol/40 µL, *n* = 7) to the peak (1.45 ± 0.41 pmol/40 µL, 167.8 ± 43.4% baseline) and then returned to the baseline (*P* = 0.012, Friedman test; Figure 4A). The peak % baseline value of extracellular GABA concentration was significantly greater than that of the control (insertion of dialysis probe without stimulation) (*P* = 0.024; Mann–Whitney *U*-test). By contrast, the extracellular concentration of glutamate (3.87 ± 0.17 pmol/40 µL; *n* = 5) did not increase during stimulation (3.37 ± 0.96 pmol/40 µL, *P* = 0.691, Mann–Whitney *U*-test; Figure 4B), confirming that excitatory synaptic transmission within the MZ is partially mediated by GABA but not by glutamate (Dammerman et al., 2000a).

In terms of endogenous glycine receptor agonists, glycine effluxes were not evoked by electrical stimulation (6.01 ± 0.37 pmol/40 µL in baseline, 5.09 ± 1.74 pmol/40 µL during stimulation, *n* = 6, *P* = 0.394, Mann–Whitney *U*-test; Figure 4C). By contrast, extracellular concentration of taurine significantly increased from the baseline (133.99 ± 4.84 pmol/40 µL, *n* = 6) to the peak (230.43 ± 53.23 pmol/40 µL, 183.7 ± 19.7%) and then gradually returned to the baseline after about 120 min (*P* = 0.0025, Friedman test; Figure 4D). The peak % baseline value of extracellular taurine concentration was significantly larger than that of the control (*P* = 0.0087; Mann–Whitney *U*-test).

### Background neuronal activity may be responsible for ambient taurine

**Figure 5:**
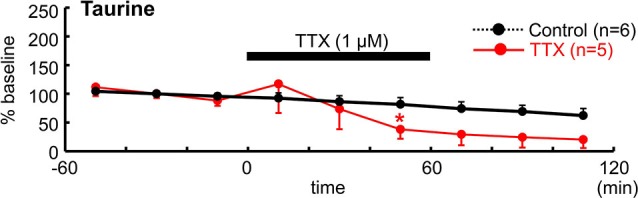
**TTX reduced ambient taurine levels**. Temporal changes in extracellular taurine concentrations after bath application of 1 µM TTX for 60 min during microdialysis were estimated and are indicated as % baseline value. TTX was applied as indicated. The baseline value was defined as the average value 60 min immediately before TTX application and the control data are from Figure 4D. ^*^P < 0.05 as compared with control (Mann–Whitney *U*-test).

To determine whether neuronal activity is responsible for the release of endogenous taurine, we treated tangential neocortical slices with TTX for 60 min during the microdialysis assay (Figure 5). Application of TTX (1 µM) significantly reduced ambient taurine levels (% baseline) in tangential neocortical slices at 50 min (control, 81.5 ± 11.9%, TTX, 38.0 ± 11.5%; *n* = 5, *P* = 0.0446; Mann–Whitney *U*-test). These results suggested that endogenous taurine is released in an activity dependent manner.

### Cellular and subcellular localization of taurine

**Figure 6:**
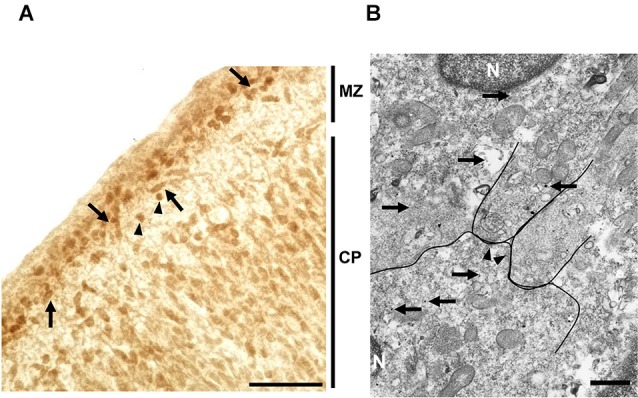
**Localization of taurine in the neonatal MZ**. **(A)** Taurine immunoreactivity was observed both in the MZ and cortical plate (CP) in coronal sections from the P0 rat forebrain. Note that both Cajal-Retzius-like cells (arrows) and non-Cajal-Retzius-like cells (arrowheads) showed taurine immunoreactivity. Scale bar: 40 µm. **(B)** Electron microscopic analysis using section immunolabeled for taurine (silver-intensified ultra-small gold particles (arrows)), indicating that taurine was intracellularly localized in cells located in the MZ. Arrows indicate taurine-immunoreactive signals. The postsynaptic density (arrowheads), nucleus (N). Note that taurine signals were not detected in presynaptic structures. Scale bar: 500 nm.

Localization of taurine in the MZ of the developing cerebral cortex was examined by immunohistochemistry. At P0, immunoreactivity for taurine was found in both Cajal-Retzius-like and non-Cajal-Retzius-like cells (Figure 6A). These results suggested that both cells in the MZ could be a source of taurine. To further identify the precise localization of taurine in these cells, we performed immuno-electron microscopic analysis with a taurine antibody. Interestingly, electron dense signals for taurine immunoreactivity were present intracellularly (Figure 6B), although these were not observed in presynaptic structures such as synaptic vesicles in the specimens that we examined. These results suggested that taurine may be released from the cell soma.

### Taurine transporter did not contribute to the spread of excitation

**Figure 7:**
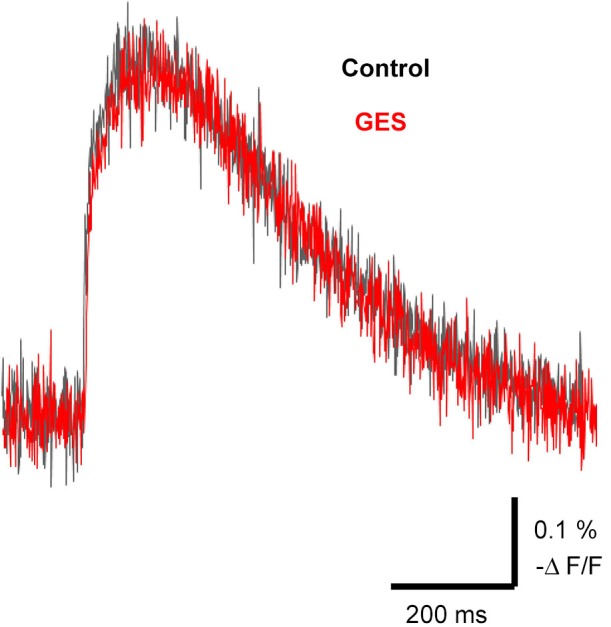
**Propagation of excitation was not affected by the taurine transporter inhibitor GES**. Typical traces of evoked optical signals with or without GES. Note that application of GES (1 mM) did not affect the amplitude of the signals (red trace). GES was applied at least for 10 min before stimulation.

It is known that external taurine stimulates taurine uptake by the Na^+^-dependent taurine transporter, inducing a membrane depolarization by the net movement of positive charge with the stoichiometric transport of 2 Na^+^: 1 Cl^−^: 1 taurine (Sarkar et al., 2003) and a long-lasting enhancement of neurotransmission (Chepkova et al., 2002; Sergeeva et al., 2003; Chepkova et al., 2006). To assess the role of taurine transporter activity in the spread of excitation in the MZ, we studied the effects of a competitive taurine transporter inhibitor, GES, on the propagation of evoked signals. The application of GES (1 mM) did not affect evoked optical signals (97.1 ± 3.9%, *n* = 4, *P* = 0.063, paired *t*-test; Figure 7). Thus, GES should have no influence on the spread of excitation in the MZ.

## Discussion

In the present study, we demonstrated that excitatory neurotransmission along the tangential axis of the MZ is mediated mainly by GABA_A_ and glycine receptors but not by glutamate receptors. In addition, we showed that GABA and the GABAergic and glycinergic agonist taurine, but not glycine or glutamate, are released upon electrical stimulation. We conclude from these observations that GABA and taurine may act as endogenous agonists of both GABA_A_ and glycine receptors (Flint et al., 1998; Kilb et al., 2002), thereby facilitating excitatory neurotransmission in the neonatal MZ.

Our present study demonstrates that TTX-sensitive neuronal activity is required for both excitatory propagation and ambient taurine release in the neonatal MZ. In contrast to the complete blockade of postsynaptic currents in the patch-clamp experiments, in optical voltage recordings a Ca^2+^-insensitive component remains, which most probably reflects stimulus-induced depolarization of presynaptic elements within the MZ. Because propagation depends on neurotransmission mediated by activation of glycine receptors as well as GABA_A_ receptors, evoked and/or activity dependent release of taurine, an endogenous agonist preferentially for glycine receptors, should play a key role in the propagation of excitation over the MZ. It has already been reported that taurine can tonically activate glycine receptors in migrating and differentiating neurons in the immature cortical plate before synaptogenesis occurs (Flint et al., 1998). Under these conditions, taurine is released in the absence of action potentials and extracellular calcium. In addition, a non-synaptic taurine release has also been suggested in the MZ of the developing neocortex (Kilb et al., 2008). Although we did not investigate the release mechanism of neuronal activity-dependent ambient taurine, our present findings shows that evoked taurine release is involved in neurotransmission in the MZ. While it is shown that taurine can be released from the immature cerebral cortex in response to depolarization or tetanic electrical stimulation (Collins and Topiwala, 1974; Oja and Saransaari, 2013), the mechanism underlying activity-dependent release of taurine remains unclear (Collins and Topiwala, 1974; Oja and Saransaari, 2013).

A dense, transient GABAergic fiber plexus in the MZ has been considered to form functional synaptic contacts to pyramidal neurons (Imamoto et al., 1994; Marín-Padilla, 1998; Dammerman et al., 2000a,b; Radnikow et al., 2002). It has been further suggested that the synapses mediated by GABA_A_ receptors in the MZ could provide localized excitatory drive onto the distal apical dendrites of immature cortical neurons thereby influencing early synaptic connections (Dammerman et al., 2000a). Our present results that excitatory synaptic transmission is mediated by GABA in the MZ are compatible with previous studies (Mienville, 1998; Schwartz et al., 1998; Aguiló et al., 1999; Dammerman et al., 2000a; Soda et al., 2003). The evidence that GABA was released by electrical stimulation also supports this interpretation.

Previous studies have demonstrated excitatory glutamatergic responses in Cajal-Retzius cells (Aguiló et al., 1999; Mienville and Pesold, 1999; Radnikow et al., 2002; Chan and Yeh, 2003) as well as in non-Cajal-Retzius cells (Schwartz et al., 1998; Soda et al., 2003). Although other glutamatergic cells may exist in the MZ (Martínez-Galán et al., 2001; Hevner et al., 2003), Cajal-Retzius cells represent a population of glutamatergic neurons, at least in the neonatal mouse (Soriano and Del Río, 2005). Consistent with our results, spontaneous glutamatergic postsynaptic potentials were not detected in Cajal-Retzius cells (Kilb and Luhmann, 2001; Quattrocolo and Maccaferri, 2013) and postsynaptic potentials was not evoked by glutamate application in the MZ (Dammerman et al., 2000a). Furthermore, whole-cell patch-clamp recordings from Cajal-Retzius cells in this study also confirmed this result. Although glutamatergic inputs may arise from deeper cortical layers (Soda et al., 2003), glutamatergic networks activity does not contribute to synaptic transmission in the MZ in a tangential direction (see Figure 1B). Accordingly, glutamate was not released by electrical stimulation which evoked the spread of excitation. This observation does not exclude the reported glutamatergic neurotransmission in the MZ (Mienville and Pesold, 1999; Martínez-Galán et al., 2001; Lu et al., 2001; Chan and Yeh, 2003; Dvorzhak et al., 2012) that may be attributable to different pathways of information transfer in this layer due to differences in animal species, age and sectioning of slices.

Previously we reported glycine receptor mediated depolarization (Kilb et al., 2002) and the functional expression of α_2_/β glycine receptors (Okabe et al., 2004) in rat Cajal-Retzius cells. A recent study suggests that glycine receptors are also involved in corticogenesis by controlling radial neuronal migration (Nimmervoll et al., 2011). However, it is so far unknown whether glycine receptor-mediated depolarization affects neurotransmission. Our present results suggest that they facilitate neurotransmission, which spreads over the MZ. The effects of the GABA_A_ and glycine receptor antagonists were additive, this result suggests that not only GABA_A_ receptors but also glycine receptors are involved in synaptic transmission in the MZ. The mechanism underlying neurotransmission mediated by glycine receptors could be different from that reported recently in the developing visual cortex, as tonic activation of presynaptic glycine receptors enhanced excitatory neurotransmitter release (Kunz et al., 2012).

Taurine has been shown to act as an endogenous agonist for glycine receptors, with a lower affinity than glycine (Flint et al., 1998; Kilb et al., 2002; Okabe et al., 2004; Le-Corronc et al., 2011). The results from microdialysis suggest that an endogenous agonist for glycine receptors should be taurine but not glycine, because taurine was released by electrical stimulation. In addition, immunohistochemistry for taurine revealed that both Cajal-Retzius-like cells and non-Cajal-Retzius-like cells contain taurine. Moreover our immunoelectron microscopic analysis revealed that taurine was present inside the cell soma, but not in synaptic structures (Figure 6). It has been reported that taurine activates not only glycine receptors but also GABA_A_ receptors (Chepkova et al., 2002; Jia et al., 2008; Le-Corronc et al., 2011). Furthermore, taurine can modulate GABA_A_-receptor mediated neurotransmission (Sergeeva et al., 2007). Thus, taurine released by stimulation might activate and/or modulate GABA_A_ receptors as well as glycine receptors.

Several reports demonstrated that taurine induces long-lasting enhancement of neurotransmission. In corticostriatal pathway, an involvement of taurine uptake by Na^+^-dependent taurine transporters accompanied by membrane depolarization has been considered (Chepkova et al., 2002; Sarkar et al., 2003; Sergeeva et al., 2003; Chepkova et al., 2006). Although such a mechanism might also facilitate the spread of excitation in the MZ, it is unlikely according to our present results, because the taurine transporter inhibitor GES had no effect on the propagation of the evoked signals. By contrast, in the hippocampus, a robust long lasting potentiation of synaptic transmission by taurine is dependent on GES-sensitive taurine transport (Galarreta et al., 1996; del Olmo et al., 2004; Dominy et al., 2004). In the present study, GES did not affect the electrical stimulation-induced excitatory propagation in the MZ; despite that, inhibiting taurine uptake can increase extracellular taurine levels. One possible explanation is that extracellular taurine increased by electrical stimulation might already reach a saturating level of facilitation in the propagation of excitation. Although the precise mechanism underlying taurine-mediated neurotransmission remains to be identified, we suggest that taurine should facilitate excitatory propagation independent of GES-mediated depolarization.

The Na^+^,K^+^-2Cl^−^ cotransporter allows Cl^−^ to accumulate in cells and contributes to higher [Cl^−^]_i_ than expected levels for passive distribution. Therefore, GABA_A_ receptor activation can be depolarizing and could be occasionally excitatory in immature neurons (Shimizu-Okabe et al., 2002; Payne et al., 2003; Yamada et al., 2004; Fukuda, 2005; Ben-Ari et al., 2012). Because inhibition of Na^+^,K^+^-2Cl^−^ cotransporters by BUM attenuated the propagation of excitation, GABA_A_ and glycine receptor-mediated actions definitely depend on depolarizing membrane responses owing to elevated [Cl^−^]_i_ levels. While Bulley et al. (2013) reported that taurine can regulate voltage-gated potassium channels by a mechanism independent of Cl^−^ channels, our results suggest that regulation of neurotransmission by taurine requires ligand-gated Cl^−^ channel opening and immature-type Cl^−^ homeostasis.

Our immunoelectron microscopic analysis demonstrated that taurine is located inside immature neurons, but was unable to detect taurine in presynaptic structures. These results imply that release of taurine may be controlled via a non-vesicular process, which is consistent with previous studies that suggest taurine can be released as osmolyte by some non-vesicular systems in neurons or glial cells (Flint et al., 1998; Mulligan and MacVicar, 2006). Because a reverse mode of taurine transport is not likely attributable in the present study, taurine might be released by anion channels or another unidentified mechanisms that are regulated by neuronal activity. The volume-sensitive anion channel is known to be activated by cellular swelling (Inoue and Okada, 2007) as well as radical oxygen species (Liu et al., 2009), both of which could be activated by neuronal activities (Takagi et al., 2002; Kann and Kovács, 2007).

In the present study, electrical stimulation-induced elevation of extracellular taurine levels persisted for longer time than that of GABA. It could be because ambient taurine increased 100 times more than GABA, consuming more time for diffusion. Alternatively, GABA is vesicularly released in the synapse and is rapidly cleared by presynaptic and perisynapse-astrocytic GABA transporters, GAT1 and GAT3, respectively (Egawa et al., 2013), whereas taurine may be released from volume-sensitive anion channels (Ando et al., 2012; Furukawa et al., 2014) and be uptaken by taurine transporter-mediated mechanism (Galarreta et al., 1996; del Olmo et al., 2004; Dominy et al., 2004). Also as discussed above, taurine is localized in the cytoplasm, not in the presynaptic structures. Considering these differences in spatial release-uptake mechanism between GABA and taurine, clearance of taurine may need much longer time than that of GABA (see Figure 4).

In conclusion, this is the first report demonstrating that endogenous taurine is functionally involved in excitatory synaptic transmission spreading horizontally within the MZ. Taurine rather than glycine could be the endogenous agonist for glycine receptors. Thus, endogenous taurine acting on glycine receptors contribute to excitatory neurotransmission that is mediated by GABA in the MZ, in which cells have higher [Cl^−^]_i_ than that expected for passive distribution. Therefore, endogenous taurine in the MZ directly influences information processing in the immature MZ, thereby possibly influencing important developmental processes, such as cell migration, axonal growth and lamination of the developing cerebral cortex.

## Conflict of interest statement

The authors declare that the research was conducted in the absence of any commercial or financial relationships that could be construed as a potential conflict of interest.
